# Refrigerators for Sick Chambers

**Published:** 1881-11

**Authors:** 


### Refrigerators for Sick Chambers.

A great deal has been said and written during the last few weeks about the means taken to preserve an equally cool temperature in the sick chamber of the President of the United

States. Those who have read the description given of the apparatus employed in this case must have perceived that it is not available for ordinary use. There is, however, a very simple method of lowering the temperature of a sick-room, and keeping it fairly equal, which is "practicable," at least in most populous localities. It consists in the placing of large blocks of ice in suitable positions, and allowing them to melt slowly. The mass must in each instance be large, and it should be placed on a stool or tripod in a tub or bath, so that the water may drain away from it as it melts slowly and regularly. If one of these blocks is placed near the principle aperture by which fresh air enters a sick chamber a cold current may be secured. If two of three large blocks are placed in advantageous positions around, though not too close to, the bed, the surrounding atmosphere will be much reduced in temperature. Unless the general heat of the chamber be great, the moisture of the air due to evaperation will be very small, and this may be almost wholly prevented by removing the ice water as quickly as the block melts. Large blocks do not, however, thaw rapidly, and they continuously absorb heat from the atmosphere, rendering a chamber of moderate dimensions easy to cool and keep cool during a lengthened period. The cost of carrying out this system of heat reduction effectually will not exceed a few shillings, and what is of primary importance, no preparation or noisy proceeding is required. The blocks of ice can be supplied by any wholesale ice merchant, and stools or tripods, and pans or baths, are to be found in every household. If the ice does not cool the air quickly enough, the process may be expedited by sprinkling the blocks with salt.—*London Lancet.*

